# Identification of lead anti-human cytomegalovirus compounds targeting MAP4K4 via machine learning analysis of kinase inhibitor screening data

**DOI:** 10.1371/journal.pone.0201321

**Published:** 2018-07-26

**Authors:** Blair L. Strang, Christopher R. M. Asquith, Hanan F. Moshrif, Catherine M-K Ho, William J. Zuercher, Hassan Al-Ali

**Affiliations:** 1 Institute for Infection & Immunity, St George’s, University of London, London, United Kingdom; 2 Structural Genomics Consortium, UNC Eshelman School of Pharmacy, University of North Carolina at Chapel Hill, Chapel Hill, North Carolina, United States of America; 3 Lineberger Comprehensive Cancer Center, University of North Carolina at Chapel Hill, Chapel Hill, North Carolina, United States of America; 4 Miami Project to Cure Paralysis, University of Miami, Miami, Florida, United States of America; 5 Department of Neurological Surgery, University of Miami, Miami, Florida, United States of America; 6 Sylvester Comprehensive Cancer Center, University of Miami, Miami, Florida, United States of America; 7 Katz Drug Discovery Center, University of Miami, Miami, Florida, United States of America; 8 Department of Medicine, University of Miami, Miami, Florida, United States of America; University of St Andrews, UNITED KINGDOM

## Abstract

Chemogenomic approaches involving highly annotated compound sets and cell based high throughput screening are emerging as a means to identify novel drug targets. We have previously screened a collection of highly characterized kinase inhibitors (Khan *et al*., Journal of General Virology, 2016) to identify compounds that increase or decrease expression of a human cytomegalovirus (HCMV) protein in infected cells. To identify potential novel anti-HCMV drug targets we used a machine learning approach to relate our phenotypic data from the aforementioned screen to kinase inhibition profiling of compounds used in this screen. Several of the potential targets had no previously reported role in HCMV replication. We focused on one potential anti-HCMV target, MAPK4K, and identified lead compounds inhibiting MAP4K4 that have anti-HCMV activity with little cellular cytotoxicity. We found that treatment of HCMV infected cells with inhibitors of MAP4K4, or an siRNA that inhibited MAP4K4 production, reduced HCMV replication and impaired detection of IE2-60, a viral protein necessary for efficient HCMV replication. Our findings demonstrate the potential of this machine learning approach to identify novel anti-viral drug targets, which can inform the discovery of novel anti-viral lead compounds.

## Introduction

Identification of viral and cellular proteins required for virus replication can be a critical step in the discovery of novel anti-viral targets. A number of genetic methods are available to screen infected cells to identify proteins required for virus replication. These include the screening of infected cells using siRNA [1–7] or CRISPR/Cas9 [8–10] and analysis of infected haploid cells treated with “gene trap” retroviruses [11–17]. In genetic experiments, knock down or knock out of a factor in a screen can directly identify the factor required for virus replication. However, the factors required for viral replication identified in these screens may not be pharmacologically tractable (“druggable”) with small molecules. Also, genetic depletion of a protein and pharmacological inhibition of a single catalytic domain in that protein may have divergent phenotypic consequences [18].

Screening collections of compounds can directly identify small molecules with anti-viral activity. However, if the target of the compound “hits” from these screens is unknown, it is not always possible to either effectively utilize medicinal chemistry to develop more effective compounds that share the same target, or not always possible to directly identify known drugs with the same target. This can be further complicated if the compounds screened display promiscuity, as is the case of most kinase inhibitor compounds. This complexity often makes it difficult to provide novel observations regarding mechanisms of virus replication from analysis of the biochemical profiles of screened compounds.

Genetic and compound screening has been extensively used to find drug targets and drugs for viruses of clinical importance that have few therapeutic options. An example of this is human cytomegalovirus (HCMV). HCMV is a prominent cause of morbidity and mortality in a number of patient populations [19]. There is currently no widely available vaccine [20] and available anti-HCMV drugs (such as ganciclovir, valganciclovir and foscarnet) have many short-comings, including toxicity and viral drug resistance [21,22]. Several anti-HCMV drugs are in clinical trials, but may have similar shortcomings to those anti-HCMV drugs currently available [23–27]. Therefore, there is a necessity to identify and develop novel anti-HCMV compounds to improve patient outcomes.

Previously, we and others have used large scale genetic screening of siRNAs to identify factors required for HCMV replication [28–31]. This approach has had limited success in identifying pharmacologically tractable anti-viral targets. Therefore, as an alternative to genetic screening for drug targets we pursued a chemogenomic approach and screened collections of kinase inhibitors to identify those with anti-HCMV activity [32–34]. Many of the active kinase inhibitors typically displayed promiscuity [32–35] and often it was not possible to efficiently mine the data from our screens to understand which inhibited kinases were driving anti-viral activity. To identify drug targets from our screening data we revisited our analysis of a screen [33] using the GlaxoSmithKline (GSK) Published Kinase Inhibitor Set (PKIS) [36] and employed a machine learning approach [37] to analyze the relationship between the phenotypic data from our screen and the kinase inhibition profiles of the compounds used in the screen. From this analysis we identified a number of potential drug targets and investigated lead compounds targeting the kinase MAP4K4, whose function in HCMV replication was unknown.

## Materials and methods

### Machine learning analysis of kinase inhibitor screening data

Each component of the machine learning analysis described in the Results section has been previously reported [37] and was carried out at the University of Miami using Support Vector Machines. Please contact Hassan Al-Ali for information on all aspects of the machine learning analysis.

### Viruses and cells

HCMV strains AD169 and Merlin (RCMV1111) [38] were generously provided by Don Coen (Harvard Medical School) and Richard Stanton (Cardiff University), respectively. Human foreskin fibroblast (HFF) cells (clone Hs29) were obtained from the American Tissue Culture Collection.

### Western blotting

HFF cells were infected at the MOI indicated in each Figure or prepared for analysis at the time of infection. After washing with PBS, cells were resuspended in Laemmli buffer containing 5% **β**-mercaptoethanol. Proteins were separated on 8% polyacrylamide gels. Membranes were probed with antibodies recognizing IE1/2, (Virusys, 1:1000 dilution), IE2 proteins (clone 5A8.2, Millipore, 1:1000 dilution), MAP4K4 (ab155583, Abcam, 1:500 dilution) and **β**-actin (SIGMA, 1:5000 dilution). All primary antibodies were detected using anti-mouse- or anti-rabbit-horseradish peroxidase (HRP) conjugated antibodies (Millipore and Cell Signaling Technology, respectively). Chemiluminescence solution (GE Healthcare) was used to detect secondary antibodies on film. Where necessary blots were striped and re-probed. Relative band intensity (band intensity relative to **β**-actin signal in the same lane) was analyzed using ImageJ software obtained from the National Institutes of Health (USA). Thusly, in lanes where relative band intensity was analyzed, densitometry was used to calculate the percentage difference in band intensity between **β**-actin bands in those lanes. The percentage difference in band intensity for specific proteins in those lanes was then calculated. Specific protein band intensity was divided by **β**-actin intensity to calculate relative band intensity.

### Treatment of cells with siRNA and infection of transfected cells

Twenty four hours before transfection 1 x 10^5^ HFF per well were seeded in 12-well plates in media with no antibiotics. siControl Non targeting siRNA #3 (D-001810-03-05) or ON-TARGETplus or SMARTpool MAP4K4 siRNA (L-003971-00-0005) (both Dharmacon/GE) were used. Per well, 113 μl of 1 μM siRNA and 2 μl Dharmafect2 (Dharmacon/GE) were diluted in 93 μl and 146 μl OptiMEM (Invitrogen), respectively. After 5 mins at room temperature, both solutions were combined. After 20 mins, media was removed from each well and replaced with the siRNA/Dharmafect mixture, then 500μl of media with no antibiotics was added to each well. Transfected cells were incubated at 37°C for 72 hours then used as indicated in the text.

### Compounds

PF06260933 dihydrochloride [39] was purchased from Bio-Techne (Minneapolis, MN, USA). Ganciclovir was purchased from SIGMA, UK). JNK-8-IN was a kind gift from Nathanael Gray (Harvard Medical School). A 4-Amino-pyridopyrimidine compound, here designated CA409, was synthesized as previously reported [40]. All compounds were resuspended in dimethyl sulfoxide (DMSO).

### Viral yield reduction assays

HFF cells (5 × 10^4^ per well) were incubated overnight and infected at an MOI of 1. Virus was adsorbed to cells for 1 hour at 37°C and then infected cells were incubated with 0.5 ml of media containing DMSO or compound at a range of concentrations in duplicate. Plates were incubated for 72 hours at 37°C. The final concentration of DMSO in all samples was maintained at <1% (v/v).Viral titre (plaque forming units (p.f.u.) per ml) was determined by titration of viral supernatants on HFF monolayers. The mean value of duplicate plaque counts was determined and the percentage of viral titre in the presence of compound compared to control was calculated. To determine ED_50_ values, parentage inhibition versus compound concentration was plotted using Microsoft Excel and a linear fit model was used to determine the concentration at which virus yield was reduced by 50%.

### MTT assays

HFF cells (1 × 10^4^ per well) were incubated overnight and then treated for 72 hours with either DMSO or compound at range of concentrations (2 fold dilution series starting at 50 **μ**M) in duplicate. Relative cell number was then determined with an MTT assay according to the manufacturer's instructions (GE Healthcare). The mean value of duplicate readings was determined and the percentage of assay output in the presence of compound compared to DMSO was calculated. The final concentration of DMSO in all samples was maintained at <1% (v/v). As a positive control, in all experiments a 2-fold dilution series of HFF cells starting at 1 × 10^4^ cells per well was included. In each experiment we found a linear relationship between the number of cells per well and output from the MTT assay (data not shown).

## Results

### Collection and organization of kinase inhibitor screening data for machine learning analysis

**Fig 1:**
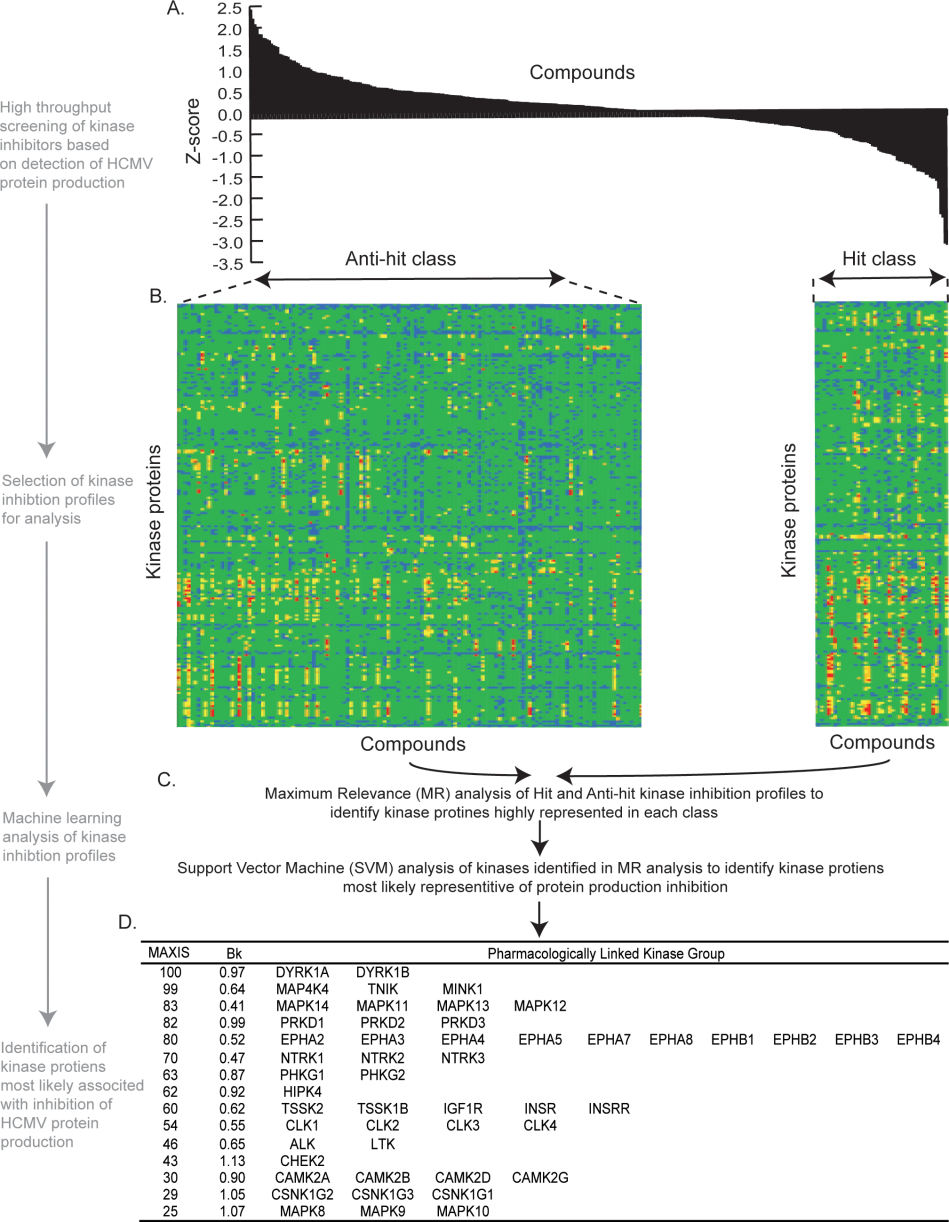
Analysis of hit and anti-hit classes of screening data. (A) z-scores from screening of GSK PKIS collection (version 1) [33], where each bar represents a single compound. (B) Heatmaps of kinase inhibition profiling of compounds grouped from Hit and Anti-Hit classes. The potency of each compound at 1**μ**M concentration against a particular kinase is represented in colour (less than 0% inhibition–blue, 0–50% inhibition–green, 51–75% inhibition–yellow, 76–90% inhibition–orange, greater than 91% inhibition–red). Each row represents a kinase tested and each column represents a compound. (C) Schematic of machine learning analysis of Hit and Anti-hit kinase inhibition profiles. (D) Table of machine learning outputs in which pharmacologically linked kinase groups are listed with their MAXIS and Bk scores. Abbreviations in the table: ALK: Anaplastic lymphoma kinase, CAMK2: Calcium/calmodulin-dependent protein kinase type II subunit, CHEK2: Checkpoint Kinase 2, CLK: CDC-like kinase, CSNK1G1: Casein Kinase 1 Gamma 1, DYRK: Dual specificity tyrosine-phosphorylation-regulated *kinase*, EPHA: Ephrin type-A receptor, HIPK4: Homeodomain Interacting Protein Kinase 4, IGF1R: Insulin-like growth factor 1 receptor, INSR: Insulin receptor, INSRR: Insulin Receptor Related Receptor, *JNK*: c-Jun N-terminal *kinase*, LTK: Leukocyte Receptor Tyrosine Kinase, MAPK: Mitogen-activated protein kinase, MAP4K4: Mitogen-Activated Protein Kinase Kinase Kinase Kinase 4, MINK1: Misshapen Like Kinase 1, NTRK: TKR receptor kinase, PHKG: Phosphorylase Kinase Catalytic Subunit Gamma, PRKD: Serine/threonine-protein kinase, TNIK: TRAF2 And NCK Interacting Kinase, TRAF2: *TNF Receptor Associated Factor 2*, TSSK: Testis Specific Serine Kinase.

Previously, we devised a cell based high throughput methodology [33] to screen the GSK PKIS collection of kinase inhibitors [35,36] for their ability to increase or decrease the expression of a viral protein, pp28, in cells infected with HCMV high passage strain AD169. After excluding screened compounds for toxicity effects [33], we interpreted the results of our screen [33] as a z-score [41,42], where a positive or negative z-score represented an increase or decrease, respectively, in the number of pp28 positive cells in the presence of each compound. The z-score for each compound is shown in Fig 1A, where each bar represents the z-score of a single compound.

The GSK PKIS collection has been extensively characterized [35], including each compounds kinase inhibition profile; biochemical analysis of each compounds ability to inhibit 224 human kinases using *in vitro* assays. The kinase inhibition profiles of PKIS compounds demonstrated that nearly every compound in this collection displayed some degree of promiscuity [33,35]. To deconvolute the kinase inhibition profiles of compounds and identify kinases that inhibit HCMV replication, we subjected our GSK PKIS screen data to a machine learning algorithm that was previously developed and validated in a mammalian screening system [37].

The kinase inhibition profiles of compounds with z-scores between <-0.75 (“hit class”) and >0.25 (“anti-hit class”) were selected. Profiles of compounds with z-scores between < -0.75 and > 0.25 were chosen to ensure a separation of at least 1 between the hit and anti-hit classes and to ensure the profiles that potentially had the most information were analyzed. The aforementioned kinase inhibition profiles are shown in Fig 1B, where the heatmap indicated the potency of kinase inhibition (each row represents a kinase tested and each column represents a compound). The selected kinase profiles in Fig 1B were analyzed using a maximum relevance (MR) algorithm [37] to identify kinases whose inhibition in both classes had the highest information content (Fig 1C). Thus, the MR analysis was able to produce a list of kinase proteins most likely related to either inhibition or promotion of HCMV protein production.

### Identification of potential drug targets within pharmacologically linked kinase groups

From the kinases selected by MR analysis, a greedy backwards feature selection algorithm using support vector machines (SVM) [37] was then used to identify the minimum number of kinases whose inhibition was highly predictive of HCMV protein production inhibition (Fig 1C). These kinases were referred to as the Maximum Information Set (MAXIS). Closely related kinases can have similar inhibition profiles, termed “pharmacological linkage”. Therefore, the MAXIS kinase proteins were grouped as pharmacologically linked kinases (Fig 1D) (Analysis of sequence homology and pharmacological similarity that identified the pharmacologically relationship between kinases has been previously described [37].) Each group was given a MAXIS score to indicate the number of times kinase proteins within each group had been analyzed by SVM [37] (Fig 1C and 1D). The greater the number of times a kinase is selected by the selection algorithm increases the MAXIS score. To determine whether kinase groups with MAXIS scores were acting as targets (inhibition resulted in suppression of HCMV protein production) or anti-targets (inhibition resulted in promotion of HCMV protein production), we used a previously developed inhibition bias metric, Bk [37]. A positive Bk score indicated that the MAXIS kinase was a candidate target, while a negative Bk score indicated that a kinase was a candidate anti-target (Fig 1D). Therefore, the analysis of our GSK PKIS screening data yielded 15 groups of pharmacologically related kinases with positive Bk scores, indicating one or more members of each groups was a potential target for inhibiting HCMV protein production (Fig 1D).

Many of the kinase proteins shown in Fig 1D had no known role in HCMV replication. To elucidate which members of each pharmacologically linked group were relevant to HCMV replication and, therefore, potential drug targets, we sought to understand which proteins were present in HCMV infected cells and which facilitated HCMV replication.

We compared proteins in each group (Fig 1D) to a proteomics dataset listing proteins that have previously been found in human fibroblasts infected with HCMV [43] (Table 1). Nearly every group contained at least one kinase protein found in this proteomic dataset. We then compared the proteins in each group to datasets in which collections of siRNAs had been used to understand the requirement for kinase proteins in HCMV replication [30] or HCMV protein production [28] (Table 1). Many of the siRNA had no obvious effect in the siRNA screen, or were toxic to infected cells in the screen.

**Table 1:** Scores of pharmacologically linked kinase proteins and analysis compared to other datasets.

MAXIS score	Kinase Group	ProteomicAnalysis^1^	siRNA Dataset 1^2^	siRNA Dataset 2^3^
100	DYRK1A	+		NC
	DYRK1B	ND	NC	T
99	MAP4K4	+	NC	NC
	TNIK	ND	NC	T
	MINK1	+	NC	NC
83	MAPK14	+	NC	T
	MAPK11	ND	NC	T
	MAPK13	+	NC	T
	MAPK12	ND	NC	
82	PRKD1	+	NC	T
	PRKD2	ND	NC	T
	PRKD3	ND	NC	T
80	EPHA2	+	NC	NC
	EPHA3	ND	NC	T
	EPHA4	+	NC	T
	EPHA5	ND	NC	T
	EPHA7	ND	NC	T
	EPHA8	ND	NC	T
	EPHB1	ND	NC	T
	EPHB2	+	NC	
	EPHB3	+	NC	T
	EPHB4	+	NC	
70	NTRK1	+	NC	NC
	NTRK2	+	NC	T
	NTRK3	+	NC	T
63	PHKG1	ND	NC	T
	PHKG2	ND	NC	T
62	HIPK4	ND	NC	T
60	TSSK2	ND	NC	T
	TSSK1B	ND	NC	T
	IGF1R	+	NC	T
	INSR	+		T
	INSRR	ND		T
54	CLK1	+	NC	T
	CLK2	+	NC	T
	CLK3	+	NC	T
	CLK4	ND	NC	T
46	ALK	ND	NC	T
	LTK	ND	NC	
43	CHEK2	+		NC
30	CAMK2A	ND	NC	T
	CAMK2B	ND	NC	NC
	CAMK2D	+	NC	T
	CAMK2G	+	NC	NC
29	CSNK1G2	ND	NC	NC
	CSNK1G3	+	NC	T
	CSNK1G1	+		T
25	MAPK8	+	NC	NC
	MAPK9	+	NC	T
	MAPK10	ND	NC	T

^1^Data from reference [43]. Plus symbol = detected, ND = not detected.

^2^Data from reference [30]. Green box = decrease in HCMV replication, red box = increase in HCMV replication, NC = no change in HCMV replication.

^3^Data from reference [28]. Green box = decrease in HCMV protein pp28 production, red box = increase in HCMV protein pp28 production, NC = no change in HCMV protein pp28 replication, T = toxic.

DYRK1A, CHEK2 and CSNK1G1 were present in the proteomic analysis and were found to be necessary for HCMV protein production or HCMV replication in siRNA screens (Table 1). It has been demonstrated that inhibitors of DYRK1A prevent HCMV replication [44]. Although CHEK2 (also known as Chk2) was found to be required for HCMV replication in one siRNA based study [30], it has also been reported that signaling involving CHEK2 is inhibited in HCMV infected cells [45]. Thus, the requirement for CHEK2 in HCMV replication was unclear. There was no other information on the requirement of CSNK1G1 in HCMV infected cells. Although inhibitors that specifically inhibit other casein kinase isozymes have been reported [46], there is no selective and potent inhibitor of CSNK1G1. Therefore, our analysis of proteins from the pharmacologically linked groups showed that a known anti-HCMV drug target, DYRK1A, could be identified. However, it was unclear if CHEK2 and CSNK1G1 could be considered as anti-HCMV targets.

### MAP4K4 was present in HCMV infected cells and was required for efficient HCMV replication and protein production

We noted that one group of kinase proteins including MAP4K4, TNIK and MINK1, had a high MAXIS score (Fig 1C). MAP4K4 and MINK1 were thought to be present in HCMV infected cells (Table 1). However, our analysis of siRNA did not indicate a role for any of these proteins in HCMV replication or identity a lead compound for any of these proteins (Table 1). Given the high MAXIS score of this group, we decided to investigate if one or more of the aforementioned proteins were necessary for HCMV replication.

TNIK was not reported to be found in HCMV infected cells (Table 1) and the functional role of MINK1 is unclear and may be restricted to T cells [47]. It has been reported that MAP4K4 is required for production of the IE proteins of another herpesvirus, Kaposi’s sarcoma herpesvirus (KSHV) [48,49]. Therefore, we focused on investigation of MAP4K4.

**Fig 2:**
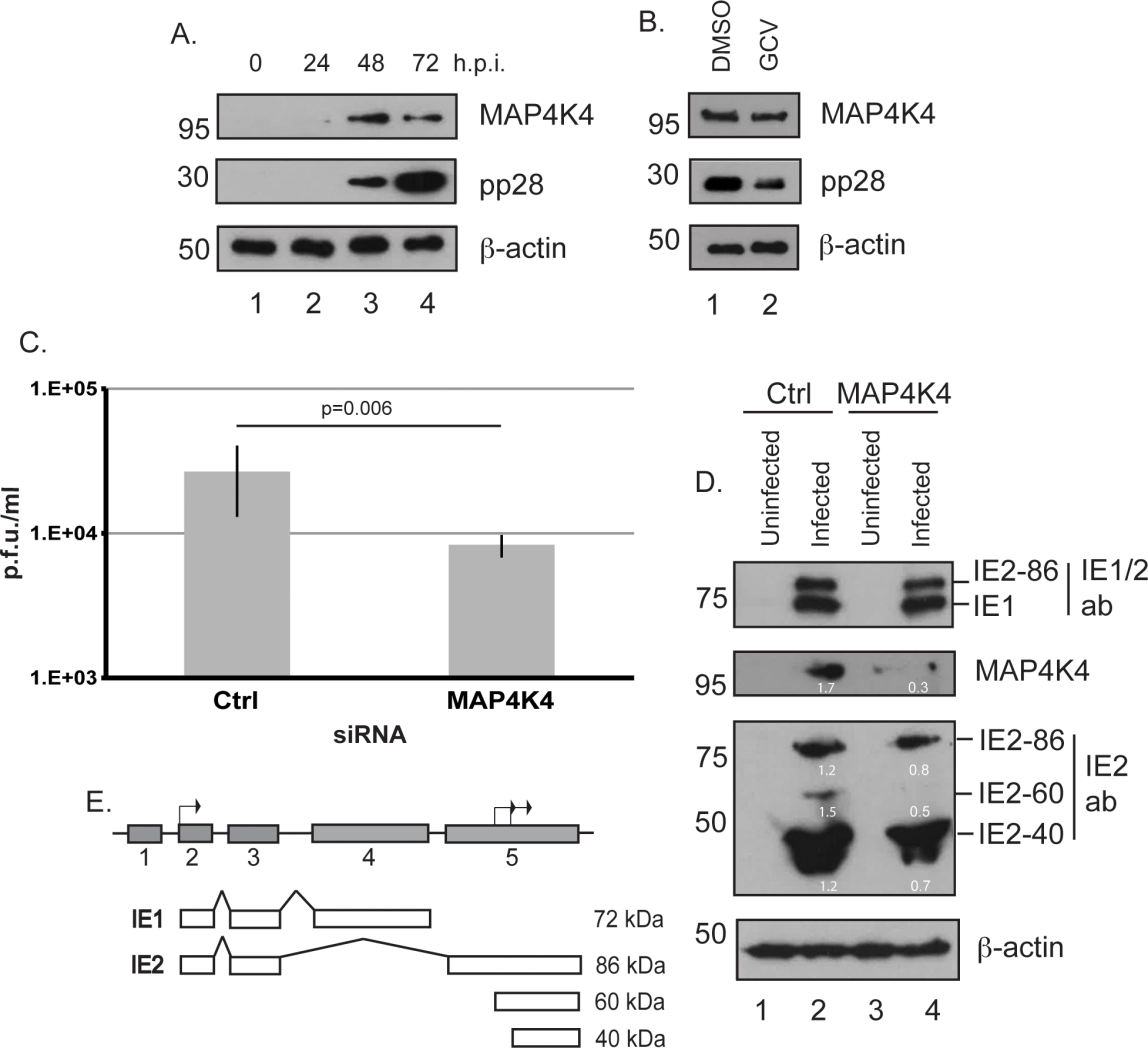
Treatment of HCMV infected cells with siRNA. (A, B and D) Western blotting of uninfected and HCMV infected cells. As outlined in the text, HFF cells were either (A) infected with AD169 (MOI of 1), (B) infected with AD169 (MOI of 1) and treated at the time of infection with either 10**μ**M ganciclovir (GCV) or the equivalent volume of DMSO, or (D) treated with siRNA and infected with AD169 (MOI of 1). Cell lysates were prepared for western blotting at the time points (hours post infection (h.p.i.)) indicated above the Figure (A) or at 72 h.p.i. (B and D). In (A) uninfected cells harvested at the time of infection are shown as 0 h.p.i.. Proteins recognized by the antibodies used are indicated to the right of each figure. Also indicated are the IE antibodies used (IE1/2, recognizing IE1 and IE2-86, and IE2, recognizing all IE2 proteins). The positions of molecular mass markers (kDa) are indicated to the left of each figure. The numbers in white represent the relative band intensity relative to the **β**-action band in the same lane. (C) Production of HCMV in cells treated with siRNA. HFF cells were treated with siRNA then infected with AD169 (MOI of 1). At 72 h.p.i. the virus released into the cell supernatant was quantified as plaque forming units (p.f.u.)/ml. The figure shows the average and standard deviation of data from three independent experiments. The result of an unpaired *t* test is shown above the data. (E) HCMV sequences encoding IE1/2 proteins and IE1/2 proteins produced during HCMV replication. Five exons of the HCMV UL122-123 locus that encode IE1 and IE2 proteins are shown in grey. Black arrows in exons 2 and 5 represent start codons. Below the exons IE1 and IE2 proteins are shown (white boxes), as are IE2 proteins IE2-60 and IE2-40 produced from internal start codons in exon 5 (white boxes). The alternative spicing of RNAs is also indicated. The molecular weight of each protein is shown to the right of the figure.

Using western blotting, we confirmed the presence of MAP4K4 in HCMV infected cells. In this assay MAP4K4 was found in HFF cells infected with AD169 at 48–72 h.p.i. (Fig 2A, lanes 3 and 4). In this and subsequent western blotting, the presence of **β**-actin was assayed to determine the amount of cell lysate in each sample. We noted that detection of MAP4K4 was co-incident with the production of the late viral protein pp28 (Fig 2A, lanes 3 and 4). Production of late viral proteins, including pp28, requires DNA replication [19]. However, in the presence of HCMV DNA replication inhibitor ganciclovir we found a decrease in pp28 production, but no obvious defect in production of MAP4K4 using western blotting (Fig 2B), indicating MAP4K4 production was not dependent on HCMV DNA synthesis.

To investigate if MAP4K4 was necessary for HCMV replication, we treated HFF cells with siRNA targeting production of MAP4K4 or a control siRNA, then challenged those cells with high passage HCMV strain AD169. Virus released into the supernatant of infected cells was quantified (Fig 2C). In parallel, AD169 infected cells treated with siRNA were prepared for western blotting to analyze the presence of viral and cellular proteins (Fig 2D). We hypothesized that in our previous siRNA screening experiments (Table 1, [28]), the concentration of siRNA targeting MAP4K4 used was too low to see effects in our screen. Therefore, in this study we increased the concentration of siRNA used in transfections by approximately 4-fold and observed no obviously harmful effects to transfected cells. Assays were carried out at 72 hours post infection, as at this time point HCMV virus production from infected cells should be underway and all HCMV proteins should be produced.

Compared to production of HCMV from cells treated with control siRNA, treatment of cells with siRNA targeting MAP4K4 production resulted in a more than 3-fold decrease in HCMV production (Fig 2C), indicating that MAP4K4 was required for efficient HCMV replication. The production of MAP4K4 was examined using western blotting. MAP4K4 was robustly detected in HCMV infected cells treated with control siRNA (Fig 2D, lane 2), but no other sample, resulting in an approximately 5-fold decrease in MAP4K4 detection (as determined by relative band intensity of bands compared to **β**-actin in the same lane) in cells treated with siRNA targeting MAP4K4 production. Further analysis of infected cells by western blotting was carried out to understand HCMV protein production. HCMV replication is dependent upon the production of Immediate Early (IE) proteins IE1 and IE2, which antagonize innate immunity and promote viral transcription, respectively. IE1 and IE2-86 are produced by alternative splicing of the same RNA (Fig 2E). At late time points, two other IE2 proteins, IE2-60 and IE2-40, are produced from translation initiation start codons in RNA from exon 5 (Fig 2E). IE2-60 and IE2-40 are essential for efficient HCMV replication [50].

Western blotting for IE proteins revealed that treatment of cells with siRNA targeting MAP4K4 production resulted in an approximately 2-fold decrease in IE2-60 and IE2-40 detection (Fig 2D), but no obvious defect in detection of either IE1 or IE2-86. Thus, a reduction in the presence of MAP4K4 in HCMV infected cells was associated with a loss of HCMV replication and impaired detection of IE2-60 and IE2-40 and a corresponding inhibition of HCMV replication.

### Lead compounds targeting MAP4K4 inhibited HCMV replication

**Fig 3:**
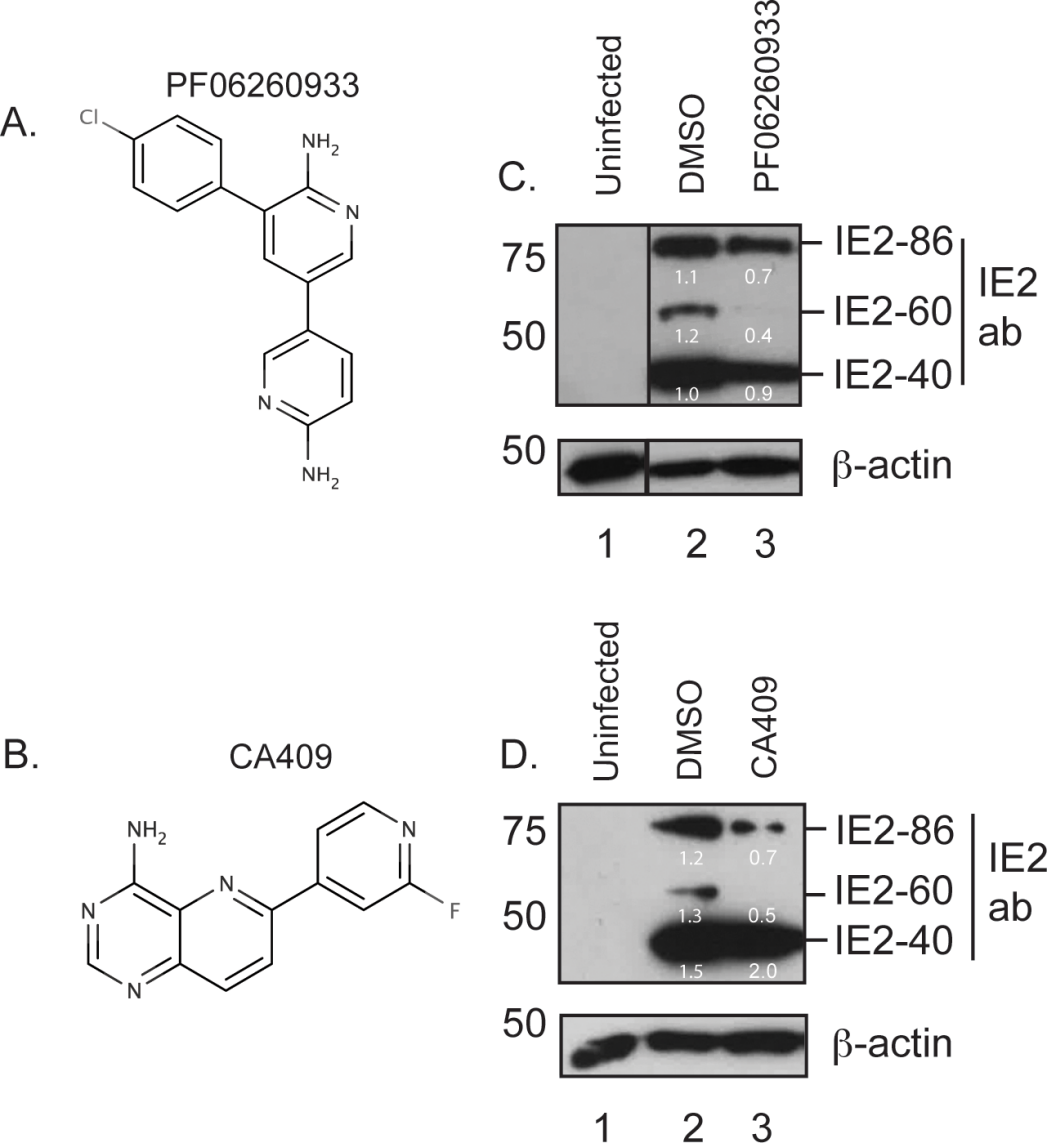
Use of PF06260933 and CA409 in HCMV infected cells. (A and B) Structure of PF06260933 and CA409, respectively. (C and D) Western blotting of PF06260933 and CA409 treated infected cells. HFF cells were infected with AD169 at an MOI of 1, then treated with either 10**μ**M PF06260933, CA409 or the equivalent volume of DMSO at the time of infection. Uninfected cell lysate (lane 1) was prepared for western blotting at the time of infection and infected cell lysate was prepared at 72 hours post infection (h.p.i.) (lanes 2 and 3). Treatment of cells is indicated above the figure. Proteins recognized by the antibodies used are indicated to the right of the figure. The positions of molecular mass markers (kDa) are indicated to the left of the figure. The numbers in white represent the relative band intensity relative to the **β**-action band in the same lane. In (B) different panels originate from the same exposure of a single membrane to film.

There has been little development of compounds targeting MAP4K4. However, we identified two structurally unrelated lead compounds, PF06260933 and CA409, reported to inhibit MAP4K4. PF06260933 (Fig 3A) strongly inhibited MAP4K4 and a number of other kinase proteins including MINK1 and TNIK [39]. CA409 (Fig 3B) was a potent and selective inhibitor of MAP4K4 and MINK1 [40].

We investigated the ability of PF06260933 and CA409 to inhibit HCMV replication in virus replication assays using the high passage HCMV strain AD169. It was observed that both PF06260933 and CA409 could inhibit HCMV replication with a 50% effective dose (ED_50_) value of approximately 10**μ**M (Table 2). To exclude the possibility that cellular cytotoxicity was responsible for the anti-HCMV effects of PF06260933 and CA409, we tested uninfected cell viability in the presence of PF06260933 and CA409 using an MTT assay to measure the activity of the mitochondrial NAD(P)H-dependent cellular oxidoreductase enzymes. We found no defect in cell viability at concentrations below 50**μ**M (Table 2), which was well above the ED_50_ value we had observed of 10**μ**M. This result indicated the anti-HCMV effects of PF06260933 and CA409 were unlikely to be due to cytotoxicity in the presence of these compounds.

**Table 2:** Anti-HCMV activity and cytotoxicity of compounds.

Compound	HCMV strain	EC_50_^1^	CC_50_^2^
PF06260933	AD169	9.6 ± 0.5	<50
CA409	AD169	12.3 ± 2.5	<50
PF06260933	Merlin(R1111)	13.3 ± 5.7	<50
CA409	Merlin(R1111)	9.6 ± 2.0	<50

^1^ 50% Effective Dose (ED_50_). Data shown is the mean ± standard deviation values (**μ**M) from three independent experiments.

^2^ 50% Cytotoxic concentration (CC_50_). Data shown is the mean value from two independent experiments (**μ**M).

Low passage strains of HCMV have a genomic content comparable to primary HCMV strains [51]. Therefore, we also tested the ability of PF06260933 and CA409 to inhibit replication of the low passage HCMV virus Merlin(R1111) [38] (Table 2). Similar results to those found when using AD169 were observed. Therefore, protein kinases inhibited by PF06260933 and CA409 were required for replication of both high and low passage HCMV viruses.

### Lead compounds targeting MAP4K4 inhibited HCMV protein production

Next, we investigated how PF06260933 and CA409 inhibited HCMV replication. Based on experiments using siRNA shown in Fig 2, we hypothesized that treatment of infected cells with inhibitors of MAP4K4 would inhibit production of IE2 proteins. Using western blotting, we assayed the production of IE2 proteins at 72 hours post infection in cells infected with AD169 and treated with the ED_50_ dose of either PF06260933 or CA409 (10**μ**M). Compared to infected cells treated with DMSO (Fig 3A, lane 2), treatment of HCMV infected cells with PF06260933 resulted in a decrease in production of all three IE2 proteins (Fig 3A, lane 3). Reduction in IE2-86 and IE2-40 production was less than 2-fold, but the reduction in IE2-60 production was approximately 2-fold. Compared to infected cells treated with DMSO (Fig 3B, lane 2), when HCMV infected cells were exposed to CA409 we observed an approximately 2-fold decrease (data not shown) in detection of IE2-86 and IE2-60 and a defect in IE2-40 production that was less than 2-fold (Fig 3B, lane 3). We found no obvious decrease in IE1 production in the presence of either PF06260933 or CA409 (data not shown). Therefore, treatment of infected cells with ED_50_ dose of either PF06260933 or CA409 resulted in an approximately 2-fold decrease in production of IE2-60.

## Discussion

We demonstrate how a machine learning approach can be applied to reveal new insights into data from high throughput compound kinase inhibitor screening. Examination of machine learning results identified potential anti-HCMV drug targets. Many of these potential targets had no previously reported roles in HCMV replication or pathogenesis. Thus, the methodology used here also has the potential to uncover hitherto unappreciated aspects of HCMV biology. Further analysis of machine learning resulted in the identification of lead compounds targeting MAP4K4 that had anti-HCMV activity.

Given the benefits of the methods we use here, we propose that our study and others will stimulate renewed interest in screening of kinase inhibitors for anti-viral effects and support the production of highly characterized kinase inhibitor collections for screening. There are, however, several points surrounding validation of machine learning results that should be addressed. We attempted to validate machine learning results by comparing our data to previously reported siRNA datasets. There was no overlap in the two datasets of siRNAs that had either positive or negative effects. However, it has been reported that there is only limited overlap in the effects of orthologous siRNAs [52]. Many of the siRNA examined in our analysis had no obvious effect in siRNA screens, or were toxic to infected cells in the screen in which they were used. The paucity of data from siRNA screens meant that several kinases could not be directly validated as drug targets. However, it is possible that the lack of effect in an siRNA screen could be the result of inefficient knockdown of protein or the statistical method of analysis used in the siRNA screening process scored an siRNA as a false negative [41,52]. Therefore, many of the kinase proteins identified in our machine learning results could be required for HCMV replication and could be anti-HCMV drug targets. Indeed, we went on to demonstrate using siRNA that MAP4K4 had effects on HCMV replication and protein production even though it had been previously reported that siRNA targeting MAP4K4 had no obvious effects in two different siRNA screens [28,30]. Thus, in future, siRNA screening data should be cautiously interpreted during validation of machine learning results.

In this study we examined the role of MAP4K4 in HCMV replication and sought to identify lead compounds targeting MAP4K4 that had anti-HCMV activity. The use of siRNA or compounds inhibiting MAP4K4 all result in a reduction in the detection of IE2-60. Thus, we propose that there is an association between the function of MAP4K4 and production of IE2-60. It is interesting to note that use of either siRNA or different compounds had different effects on production of IE2-86 and IE2-40. We propose that this may be due to different off-target effects or lack of potency of the siRNA and compounds we have used. Our observation using siRNA that knockdown of MAP4K4 leads to a reduction in IE2-60 and IE2-40 production is consistent with the somewhat limited reduction in HCMV replication in the presence of siRNA targeting MAP4K4 production. IE2-60 and IE2-40 are not essential for HCMV replication, but their expression is required for optimal HCMV replication [50]. Therefore, it is perhaps to be expected that loss of either IE2-60 or IE2-40 did not lead to a drastic reduction in HCMV replication. This leads to the question of should factors non-essential for virus replication be targeted in anti-viral strategies? We would argue that this should be considered, as there has been previous success in targeting proteins non-essential for HCMV replication. For example, the HCMV kinase protein UL97 is non-essential for HCMV replication [53], but an inhibitor of UL97, maribavir, has been used in phase III clinical trials in humans [54].

Our screen of GSK compounds was based upon inhibition of HCMV pp28 production [33]. However, it has been noted that deletion of IE2-60 or IE2-40 from the HCMV genome had no effect on pp28 production [50]. Thus, we propose that the machine learning approach used here is able to identify factors required for virus replication that were not directly related to production of pp28. We suggest that in our screen compounds that were assigned negative z-scores had inhibition profiles that contained MAP4K4 and kinase proteins that were able to inhibit pp28 production.

It remains unknown how inhibition of MAP4K4 leads to a reduction in production of IE2 proteins, as there is little understanding of MAP4K4 function. A canonical view of MAP4K4 signaling in human cells involves activation of a phosphorylation cascade that includes MAP4K4 which results in that leads to activation of the kinase JNK1 and transcriptional activation [55,56]. This may involve upstream regulation of MAP4K4 by TRAF2 [55]. We have observed an increase in TRAF2 production late in HCMV replication, similar to that which we observed with MAP4K4 in this study (data not shown). It has been reported that activation of JNK1 is inhibited in HCMV infected cells [57]. However, JNK proteins JNK1-3 (MAPK8-10) were identified in our machine learning analysis (Fig 1C). We treated HCMV infected cells with a potent inhibitor of JNK1-3, JNK-IN-8 [58], and found that this compound had very little or no effect on production of infectious HCMV (data not shown). Thus, activation of signaling that leads to JNK1 function, including that involving MAP4K4, was unlikely to be required for HCMV replication.

Other intracellular signaling pathways involving MAP4K4 have been reported [55,56], but are less well characterized. These data suggest STAT3 and NF-κB proteins are substrates of MAP4K4 [55,56]. However, we have previously demonstrated that neither canonical nor non-canonical NF-κB signaling was active in HCMV infected cells [59]. While inhibition of STAT3 can influence HCMV replication [60], we found that treatment of HCMV infected cells with either PF06260933 or CA409 had no obvious effect on STAT3 phosphorylation (data not shown). Thus, it was unlikely that inhibition of MAP4K4 in our experiments was related to the function of either STAT3 or NF-κB proteins.

Emerging evidence places MAP4K4 directly or indirectly in a number of other intracellular signaling pathways in a number of human pathologies [55,56]. Thus, it is possible that further study of MAP4K4 will uncover poorly understood, or as yet unrecognized, intracellular signaling pathways required for HCMV replication. Alternatively, we hypothesize that IE2-60 could have been a substrate of MAP4K4 in HCMV infected cells and lack of phosphorylation could have resulted in lack of protein production or detection during western blotting. As it is unclear what dictates how a protein serves as a substrate for MAP4K4. Further study of HCMV infected cells could reveal novel insights into a protein that appears to be widely used in a number of contexts [55,56].

We identify PF06260933 and CA409 as lead compounds that could be developed to be become highly active anti-HCMV compounds. This will be necessary as the ED_50_ for both compounds were in the high micromolar concentrations range despite high affinity on target results in *in vitro* binding assays [39,40]. It is possible that the weak ED_50_ of both PF06260933 and CA409 could be attributed to poor solubility, poor cell permeability and the dynamic environment in HCMV infected cells. We observed MAP4K4 production increased over time. However, we found no obvious decrease in MAP4K4 production in HCMV infected cells treated with either PF06260933 or CA409 (data not shown). This suggested that inhibition of MAP4K4 had no effect on MAP4K4 production. Regardless, these observations imply that production of proteins thought to be novel drug targets in HCMV infected cells should be assayed to investigate a potential relationship between production of protein and anti-viral effects of a compound. We argue that increased production of a protein thought to be a drug target in infected cells should not preclude development of compounds against that target, as many effective anti-viral compounds target viral proteins whose production increases over time.

Furthermore, we argue that the seemingly high ED_50_ concentrations recorded here for PF06260933 and CA409 should not preclude the development of these compounds. It is not unusual that lead compounds have somewhat high ED_50_ values before development using medical chemistry approaches. Medicinal chemistry approaches to modifying CA409 have been reported [61] and may have potential to produce a novel anti-HCMV compound with a more potent ED_50_ value. Also, maribavir, an HCMV inhibitor that has been used in human clinical trials [54], can display ED_50_ values in excess of 10**μ**M in virus yield reduction assays [62]. Thus, there is precedent for continued study of compounds that otherwise might be discarded due to somewhat limited performance in anti-viral assays.

## References

[1] Fusco DN, Brisac C, John SP, Huang YW, Chin CR, Xie T, et al (2013) A genetic screen identifies interferon-alpha effector genes required to suppress hepatitis C virus replication. Gastroenterology 144: 1438–1449, 1449 e1431-1439. 10.1053/j.gastro.2013.02.026 23462180 PMC3665646

[2] Zhu J, Gaiha GD, John SP, Pertel T, Chin CR, Gao G, et al (2012) Reactivation of latent HIV-1 by inhibition of BRD4. Cell Rep 2: 807–816. 10.1016/j.celrep.2012.09.008 23041316 PMC3523124

[3] Valle-Casuso JC, Di Nunzio F, Yang Y, Reszka N, Lienlaf M, Arhel N, et al (2012) TNPO3 is required for HIV-1 replication after nuclear import but prior to integration and binds the HIV-1 core. J Virol 86: 5931–5936. 10.1128/JVI.00451-12 22398280 PMC3347269

[4] Li Q, Brass AL, Ng A, Hu Z, Xavier RJ, Liang TJ, et al (2009) A genome-wide genetic screen for host factors required for hepatitis C virus propagation. Proc Natl Acad Sci U S A 106: 16410–16415. 10.1073/pnas.0907439106 19717417 PMC2752535

[5] Brass AL, Huang IC, Benita Y, John SP, Krishnan MN, Feeley EM, et al (2009) The IFITM proteins mediate cellular resistance to influenza A H1N1 virus, West Nile virus, and dengue virus. Cell 139: 1243–1254. 10.1016/j.cell.2009.12.017 20064371 PMC2824905

[6] Krishnan MN, Ng A, Sukumaran B, Gilfoy FD, Uchil PD, Sultana H, et al (2008) RNA interference screen for human genes associated with West Nile virus infection. Nature 455: 242–245. 10.1038/nature07207 18690214 PMC3136529

[7] Brass AL, Dykxhoorn DM, Benita Y, Yan N, Engelman A, Xavier RJ, et al (2008) Identification of host proteins required for HIV infection through a functional genomic screen. Science 319: 921–926. 10.1126/science.1152725 18187620

[8] Park RJ, Wang T, Koundakjian D, Hultquist JF, Lamothe-Molina P, Monel B, et al (2017) A genome-wide CRISPR screen identifies a restricted set of HIV host dependency factors. Nat Genet 49: 193–203. 10.1038/ng.3741 27992415 PMC5511375

[9] Heaton BE, Kennedy EM, Dumm RE, Harding AT, Sacco MT, Sachs D, et al (2017) A CRISPR Activation Screen Identifies a Pan-avian Influenza Virus Inhibitory Host Factor. Cell Rep 20: 1503–1512. 10.1016/j.celrep.2017.07.060 28813663 PMC5568676

[10] Hultquist JF, Schumann K, Woo JM, Manganaro L, McGregor MJ, Doudna J, et al (2016) A Cas9 Ribonucleoprotein Platform for Functional Genetic Studies of HIV-Host Interactions in Primary Human T Cells. Cell Rep 17: 1438–1452. 10.1016/j.celrep.2016.09.080 27783955 PMC5123761

[11] Raaben M, Jae LT, Herbert AS, Kuehne AI, Stubbs SH, Chou YY, et al (2017) NRP2 and CD63 Are Host Factors for Lujo Virus Cell Entry. Cell Host Microbe 22: 688–696 e685. 10.1016/j.chom.2017.10.002 29120745 PMC5821226

[12] Hoffmann HH, Schneider WM, Blomen VA, Scull MA, Hovnanian A, Brummelkamp TR, et al (2017) Diverse Viruses Require the Calcium Transporter SPCA1 for Maturation and Spread. Cell Host Microbe 22: 460–470 e465. 10.1016/j.chom.2017.09.002 29024641 PMC5952603

[13] Riblett AM, Blomen VA, Jae LT, Altamura LA, Doms RW, Brummelkamp TR, et al (2015) A Haploid Genetic Screen Identifies Heparan Sulfate Proteoglycans Supporting Rift Valley Fever Virus Infection. J Virol 90: 1414–1423. 10.1128/JVI.02055-15 26581979 PMC4719632

[14] Kleinfelter LM, Jangra RK, Jae LT, Herbert AS, Mittler E, Stiles KM, et al (2015) Haploid Genetic Screen Reveals a Profound and Direct Dependence on Cholesterol for Hantavirus Membrane Fusion. MBio 6: e00801 10.1128/mBio.00801-15 26126854 PMC4488941

[15] Jae LT, Raaben M, Herbert AS, Kuehne AI, Wirchnianski AS, Soh TK, et al (2014) Virus entry. Lassa virus entry requires a trigger-induced receptor switch. Science 344: 1506–1510. 10.1126/science.1252480 24970085 PMC4239993

[16] Carette JE, Raaben M, Wong AC, Herbert AS, Obernosterer G, Mulherkar N, et al (2011) Ebola virus entry requires the cholesterol transporter Niemann-Pick C1. Nature 477: 340–343. 10.1038/nature10348 21866103 PMC3175325

[17] Carette JE, Guimaraes CP, Varadarajan M, Park AS, Wuethrich I, Godarova A, et al (2009) Haploid genetic screens in human cells identify host factors used by pathogens. Science 326: 1231–1235. 10.1126/science.1178955 19965467

[18] Weiss WA, Taylor SS, Shokat KM (2007) Recognizing and exploiting differences between RNAi and small-molecule inhibitors. Nat Chem Biol 3: 739–744. 10.1038/nchembio1207-739 18007642 PMC2924165

[19] Mocarski ES, Shenk T, Griffiths PD, Pass RF (2015) Cytomegaloviruses In: Knipe DM, Howley PM, editors. Fields Virology. 6th ed. New York, NY: Lippincott, Williams & Wilkins pp. 1960–2015.

[20] Krause PR, Bialek SR, Boppana SB, Griffiths PD, Laughlin CA, Ljungman P, et al (2013) Priorities for CMV vaccine development. Vaccine 32: 4–10. 10.1016/j.vaccine.2013.09.042 24129123 PMC4623576

[21] Coen DM, Schaffer PA (2003) Antiherpesvirus drugs: a promising spectrum of new drugs and drug targets. Nat Rev Drug Discov 2: 278–288. 10.1038/nrd1065 12669027

[22] Biron KK (2006) Antiviral drugs for cytomegalovirus diseases. Antiviral Res 71: 154–163. 10.1016/j.antiviral.2006.05.002 16765457

[23] Chou S (2015) Rapid In Vitro Evolution of Human Cytomegalovirus UL56 Mutations That Confer Letermovir Resistance. Antimicrob Agents Chemother 59: 6588–6593. 10.1128/AAC.01623-15 26259791 PMC4576131

[24] Chou S (2017) A third component of the human cytomegalovirus terminase complex is involved in letermovir resistance. Antiviral Res 148: 1–4. 10.1016/j.antiviral.2017.10.019 29107686 PMC5687998

[25] Chou S (2017) Comparison of Cytomegalovirus Terminase Gene Mutations Selected after Exposure to Three Distinct Inhibitor Compounds. Antimicrob Agents Chemother 61. 10.1128/AAC.01325-17 PMC5655092 28827420

[26] Chou S, Marousek GI, Senters AE, Davis MG, Biron KK (2004) Mutations in the human cytomegalovirus UL27 gene that confer resistance to maribavir. J Virol 78: 7124–7130. 10.1128/JVI.78.13.7124-7130.2004 15194788 PMC421656

[27] Chou S, Wechel LC, Marousek GI (2007) Cytomegalovirus UL97 kinase mutations that confer maribavir resistance. J Infect Dis 196: 91–94. 10.1086/518514 17538888

[28] Polachek WS, Moshrif HF, Franti M, Coen DM, Sreenu VB, Strang BL (2016) High-Throughput Small Interfering RNA Screening Identifies Phosphatidylinositol 3-Kinase Class II Alpha as Important for Production of Human Cytomegalovirus Virions. J Virol 90: 8360–8371. 10.1128/JVI.01134-16 27412598 PMC5008103

[29] Koyuncu E, Purdy JG, Rabinowitz JD, Shenk T (2013) Saturated very long chain fatty acids are required for the production of infectious human cytomegalovirus progeny. PLoS Pathog 9: e1003333 10.1371/journal.ppat.1003333 23696731 PMC3656100

[30] Terry LJ, Vastag L, Rabinowitz JD, Shenk T (2012) Human kinome profiling identifies a requirement for AMP-activated protein kinase during human cytomegalovirus infection. Proc Natl Acad Sci U S A 109: 3071–3076. 10.1073/pnas.1200494109 22315427 PMC3286917

[31] Lin YT, Prendergast J, Grey F (2017) The host ubiquitin-dependent segregase VCP/p97 is required for the onset of human cytomegalovirus replication. PLoS Pathog 13: e1006329 10.1371/journal.ppat.1006329 28494016 PMC5426786

[32] Strang BL (2017) RO0504985 is an inhibitor of CMGC kinase proteins and has anti-human cytomegalovirus activity. Antiviral Res 144: 21–26. 10.1016/j.antiviral.2017.05.004 28501424 PMC9233920

[33] Khan AS, Murray MJ, Ho CMK, Zuercher WJ, Reeves MB, Strang BL (2017) High-throughput screening of a GlaxoSmithKline protein kinase inhibitor set identifies an inhibitor of human cytomegalovirus replication that prevents CREB and histone H3 post-translational modification. J Gen Virol 98: 754–768. 10.1099/jgv.0.000713 28100301 PMC5817216

[34] Beelontally R, Wilkie GS, Lau B, Goodmaker CJ, Ho CM, Swanson CM, et al (2017) Identification of compounds with anti-human cytomegalovirus activity that inhibit production of IE2 proteins. Antiviral Res 138: 61–67. 10.1016/j.antiviral.2016.12.006 27956134 PMC5244968

[35] Elkins JM, Fedele V, Szklarz M, Abdul Azeez KR, Salah E, Mikolajczyk J, et al (2016) Comprehensive characterization of the Published Kinase Inhibitor Set. Nat Biotechnol 34: 95–103. 10.1038/nbt.3374 26501955

[36] Drewry DH, Willson TM, Zuercher WJ (2014) Seeding collaborations to advance kinase science with the GSK Published Kinase Inhibitor Set (PKIS). Curr Top Med Chem 14: 340–342. 10.2174/1568026613666131127160819 24283969 PMC4435035

[37] Al-Ali H, Lee DH, Danzi MC, Nassif H, Gautam P, Wennerberg K, et al (2015) Rational Polypharmacology: Systematically Identifying and Engaging Multiple Drug Targets To Promote Axon Growth. ACS Chem Biol 10: 1939–1951. 10.1021/acschembio.5b00289 26056718 PMC4899818

[38] Stanton RJ, Baluchova K, Dargan DJ, Cunningham C, Sheehy O, Seirafian S, et al (2010) Reconstruction of the complete human cytomegalovirus genome in a BAC reveals RL13 to be a potent inhibitor of replication. J Clin Invest 120: 3191–3208. 10.1172/JCI42955 20679731 PMC2929729

[39] Ammirati M, Bagley SW, Bhattacharya SK, Buckbinder L, Carlo AA, Conrad R, et al (2015) Discovery of an in Vivo Tool to Establish Proof-of-Concept for MAP4K4-Based Antidiabetic Treatment. ACS Med Chem Lett 6: 1128–1133. 10.1021/acsmedchemlett.5b00215 26617966 PMC4645242

[40] Crawford TD, Ndubaku CO, Chen H, Boggs JW, Bravo BJ, Delatorre K, et al (2014) Discovery of selective 4-Amino-pyridopyrimidine inhibitors of MAP4K4 using fragment-based lead identification and optimization. J Med Chem 57: 3484–3493. 10.1021/jm500155b 24673130

[41] Birmingham A, Selfors LM, Forster T, Wrobel D, Kennedy CJ, Shanks E, et al (2009) Statistical methods for analysis of high-throughput RNA interference screens. Nat Methods 6: 569–575. 10.1038/nmeth.1351 19644458 PMC2789971

[42] Zhang JH, Chung TD, Oldenburg KR (1999) A Simple Statistical Parameter for Use in Evaluation and Validation of High Throughput Screening Assays. J Biomol Screen 4: 67–73. 10.1177/108705719900400206 10838414

[43] Weekes MP, Tomasec P, Huttlin EL, Fielding CA, Nusinow D, Stanton RJ, et al (2014) Quantitative temporal viromics: an approach to investigate host-pathogen interaction. Cell 157: 1460–1472. 10.1016/j.cell.2014.04.028 24906157 PMC4048463

[44] Hutterer C, Milbradt J, Hamilton S, Zaja M, Leban J, Henry C, et al (2017) Inhibitors of dual-specificity tyrosine phosphorylation-regulated kinases (DYRK) exert a strong anti-herpesviral activity. Antiviral Res 143: 113–121. 10.1016/j.antiviral.2017.04.003 28400201

[45] Gaspar M, Shenk T (2006) Human cytomegalovirus inhibits a DNA damage response by mislocalizing checkpoint proteins. Proc Natl Acad Sci U S A 103: 2821–2826. 10.1073/pnas.0511148103 16477038 PMC1413835

[46] Huang H, Acquaviva L, Berry V, Bregman H, Chakka N, O'Connor A, et al (2012) Structure-Based Design of Potent and Selective CK1gamma Inhibitors. ACS Med Chem Lett 3: 1059–1064. 10.1021/ml300278f 24900428 PMC4025826

[47] Martinez GJ (2017) MINK1: The missing link between ROS and its inhibition of Th17 cells. J Exp Med 214: 1205–1206. 10.1084/jem.20170571 28420734 PMC5413342

[48] Haas DA, Bala K, Busche G, Weidner-Glunde M, Santag S, Kati S, et al (2013) The inflammatory kinase MAP4K4 promotes reactivation of Kaposi's sarcoma herpesvirus and enhances the invasiveness of infected endothelial cells. PLoS Pathog 9: e1003737 10.1371/journal.ppat.1003737 24244164 PMC3820715

[49] Tan X, Gao Y, Nan Y, Zhang J, Di C, Wang X, et al (2015) Cellular MicroRNA Let-7a Suppresses KSHV Replication through Targeting MAP4K4 Signaling Pathways. PLoS One 10: e0132148 10.1371/journal.pone.0132148 26197270 PMC4511191

[50] White EA, Del Rosario CJ, Sanders RL, Spector DH (2007) The IE2 60-kilodalton and 40-kilodalton proteins are dispensable for human cytomegalovirus replication but are required for efficient delayed early and late gene expression and production of infectious virus. J Virol 81: 2573–2583. 10.1128/JVI.02454-06 17202222 PMC1865986

[51] Wilkinson GW, Davison AJ, Tomasec P, Fielding CA, Aicheler R, Murrell I, et al (2015) Human cytomegalovirus: taking the strain. Med Microbiol Immunol 204: 273–284. 10.1007/s00430-015-0411-4 25894764 PMC4439430

[52] Zhu J, Davoli T, Perriera JM, Chin CR, Gaiha GD, John SP, et al (2014) Comprehensive identification of host modulators of HIV-1 replication using multiple orthologous RNAi reagents. Cell Rep 9: 752–766. 10.1016/j.celrep.2014.09.031 25373910 PMC4926641

[53] Prichard MN, Gao N, Jairath S, Mulamba G, Krosky P, Coen DM, et al (1999) A recombinant human cytomegalovirus with a large deletion in UL97 has a severe replication deficiency. J Virol 73: 5663–5670. 10364316 10.1128/jvi.73.7.5663-5670.1999 PMC112625

[54] Marty FM, Ljungman P, Papanicolaou GA, Winston DJ, Chemaly RF, Strasfeld L, et al (2011) Maribavir prophylaxis for prevention of cytomegalovirus disease in recipients of allogeneic stem-cell transplants: a phase 3, double-blind, placebo-controlled, randomised trial. Lancet Infect Dis 11: 284–292. 10.1016/S1473-3099(11)70024-X 21414843

[55] Gao X, Gao C, Liu G, Hu J (2016) MAP4K4: an emerging therapeutic target in cancer. Cell Biosci 6: 56 10.1186/s13578-016-0121-7 27800153 PMC5084373

[56] Virbasius JV, Czech MP (2016) Map4k4 Signaling Nodes in Metabolic and Cardiovascular Diseases. Trends Endocrinol Metab 27: 484–492. 10.1016/j.tem.2016.04.006 27160798 PMC4912878

[57] Xuan B, Qian Z, Torigoi E, Yu D (2009) Human cytomegalovirus protein pUL38 induces ATF4 expression, inhibits persistent JNK phosphorylation, and suppresses endoplasmic reticulum stress-induced cell death. J Virol 83: 3463–3474. 10.1128/JVI.02307-08 19193809 PMC2663240

[58] Zhang T, Inesta-Vaquera F, Niepel M, Zhang J, Ficarro SB, Machleidt T, et al (2012) Discovery of potent and selective covalent inhibitors of JNK. Chem Biol 19: 140–154. 10.1016/j.chembiol.2011.11.010 22284361 PMC3270411

[59] Ho CM, Donovan-Banfield IZ, Tan L, Zhang T, Gray NS, Strang BL (2016) Inhibition of IKKalpha by BAY61-3606 Reveals IKKalpha-Dependent Histone H3 Phosphorylation in Human Cytomegalovirus Infected Cells. PLoS One 11: e0150339 10.1371/journal.pone.0150339 26930276 PMC4773098

[60] Reitsma JM, Terhune SS (2013) Inhibition of cellular STAT3 synergizes with the cytomegalovirus kinase inhibitor maribavir to disrupt infection. Antiviral Res 100: 321–327. 10.1016/j.antiviral.2013.09.011 24070820 PMC3845884

[61] Ndubaku CO, Crawford TD, Chen H, Boggs JW, Drobnick J, Harris SF, et al (2015) Structure-Based Design of GNE-495, a Potent and Selective MAP4K4 Inhibitor with Efficacy in Retinal Angiogenesis. ACS Med Chem Lett 6: 913–918. 10.1021/acsmedchemlett.5b00174 26288693 PMC4538449

[62] Chou S, Van Wechel LC, Marousek GI (2006) Effect of cell culture conditions on the anticytomegalovirus activity of maribavir. Antimicrob Agents Chemother 50: 2557–2559. 10.1128/AAC.00207-06 16801445 PMC1489798

